# Gene flow, population growth and a novel substitution rate estimate in a subtidal rock specialist, the black‐faced blenny *Tripterygion delaisi* (Perciformes, Blennioidei, Tripterygiidae) from the Adriatic Sea

**DOI:** 10.1111/jzs.12110

**Published:** 2015-10-05

**Authors:** Stephan Koblmüller, Bernd Steinwender, Sara Weiß, Kristina M. Sefc

**Affiliations:** ^1^ Institute of ZoologyUniversity of GrazGrazAustria

**Keywords:** Expansion dating, Mediterranean, population expansion, sea level change, triplefin

## Abstract

Population histories depend on the interplay between exogeneous and endogeneous factors. In marine species, phylogeographic and demographic patterns are often shaped by sea level fluctuations, water currents and dispersal ability. Using mitochondrial control region sequences (*n* = 120), we infer phylogeographic structure and historic population size changes of a common littoral fish species, the black‐faced blenny *Tripterygion delaisi* (Perciformes, Blennioidei, Tripterygiidae) from the north‐eastern Adriatic Sea. We find that Adriatic *T. delaisi* are differentiated from conspecific populations in the remaining Mediterranean, but display little phylogeographic structure within the Adriatic basin. The pattern is consistent with passive dispersal of planktonic larvae along cyclonic currents within the Adriatic Sea, but limited active dispersal of adults. Demographic reconstructions are consistent with recent population expansion, probably triggered by rising sea levels after the last glacial maximum (LGM). Placing the onset of population growth between the LGM and the warming of surface waters (18 000–13 000 years BP) and employing a novel expansion dating approach, we inferred a substitution rate of 2.61–3.61% per site per MY. Our study is one of only few existing investigations of the genetic structure of animals within the Adriatic basin and is the first to provide an estimate for mitochondrial control region substitution rates in blennioid fishes.

## Introduction

**Figure 1:**
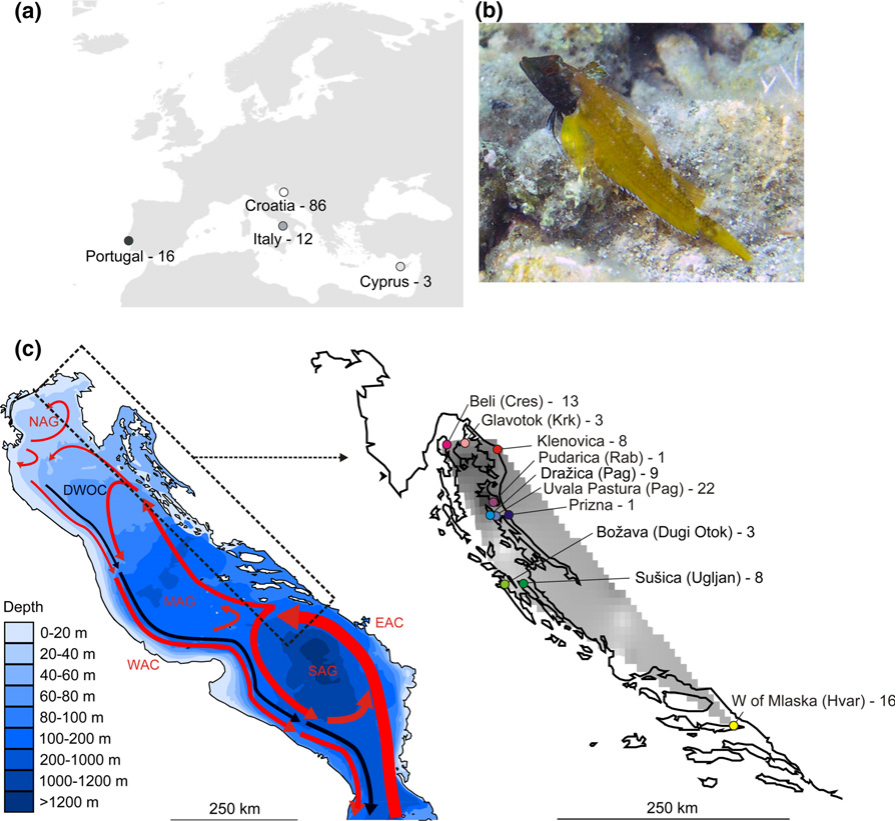
Sampling localities of *Tripterygion delaisi* used in the present study, a representative picture of a male individual *T. delaisi* and genetic landscape shape visualization. (a) Sampling locations in the Mediterranean. Sequences from Portugal, Italy, Cyprus as well as two sequences from Croatia (exact sampling locality unknown) and the out‐group sequences from the Azores and Canary islands (not shown in the map) are from Domingues et al. (2007). (b) A typical territorial male *T. delaisi*. (c) Bathymetric map of the Adriatic Sea with major currents (left) and sampling sites along the Croatian coast and islands (right), overlaid by the genetic landscape as inferred from the genetic landscape shape interpolation analysis (the degree of shading reflects genetic distance) of Adriatic *T. delaisi*. EAC, eastern Adriatic current; WAC, western Adriatic current; DWOC, deepwater outflow current; NAG, north Adriatic gyre; MAG, middle Adriatic gyre; SAG, south Adriatic gyre. For island populations, the island names are given in parentheses. Numbers to the right of the location name indicate sample size.

Environmental factors such as geology, hydrology and climate can interact strongly with ecological, morphological and reproductive characteristics of species in shaping their phylogeographic structure. For example, the glacial cycles in the Quaternary caused dramatic environmental changes that severely influenced the distribution, phylogeographic structure and demography of both aquatic and terrestrial taxa (Hewitt 2000). In marine habitats, the sea level fluctuations associated with glacial cycles had a strong impact on shelf regions and peripheral seas, and have particularly affected the demography and phylogeographic patterns of shallow water taxa, especially of those with low active dispersal capacity (e.g. Palumbi 1994; Hellberg 2009). Another important determinant of phylogeographic structure in the marine environment are water currents, which provide for dispersal during planktonic life stages (e.g. Cowen and Sponaugle 2009; Shanks 2009). The Adriatic Sea, a part of the Mediterranean Sea, is one of the marine regions, in which both sea level fluctuations and water currents may have shaped current population genetic and phylogeographic patterns to a great extent. It is connected to the rest of the Mediterranean Sea by the narrow Otranto Strait (Fig. 1) and features characteristic salinity, temperature, depth, circulation and productivity patterns (Astraldi et al. 1999). Circulation patterns in the Adriatic are dominated by cyclonic gyres, which may interrupt planktonic dispersal both within the Adriatic basin and across the Otranto Strait. Large parts of the Adriatic Sea were dry during the last glacial maximum (LGM; ~18 ka BP) when the sea level was ~120 m below its present level (Siddall et al. 2003). Subsequently, rising sea levels shifted the coastline northwards and expanded the distribution ranges of Adriatic taxa considerably (Lambeck and Purcell 2005). This rapid range expansion should have left detectable traces of population growth, especially in taxa with no or little gene flow to and from the rest of the Mediterranean Sea (Debes et al. 2008; Luttikhuizen et al. 2008).

The black‐faced blenny *Tripterygion delaisi* Cadenat & Blache 1969 (Perciformes, Blennioidei, Tripterygiidae) is a common littoral fish species in the Mediterranean Sea and the eastern Atlantic from Senegal north to the British Isles, including the Azores and Canary islands. It inhabits rocky habitats at depths of 3–40 m (Wirtz 1978) and can be observed close to the water surface only at very shadowy places (S. Koblmüller and K. M. Sefc, personal observation). Adults have a reduced swim bladder and are not able to traverse long distances across open water, which may turn, for example, larger stretches of sandy habitat into serious dispersal barriers. This low active dispersal capacity, together with high levels of territoriality in males and a well‐developed homing behaviour over short distances (Heymer 1977; Wirtz 1978), is a precondition for pronounced geographic population structure. However, in contrast to the rather stationary adult life stage, dispersal may occur during the planktonic larval phase that lasts for 16–21 days (Raventós and Macpherson 2001). While the duration of the planktonic stage would allow for long‐distance dispersal, high levels of self‐recruitment indicate that a significant proportion of larvae remain near their natal area (Carreras‐Carbonell et al. 2007), and indeed, larvae are predominantly found in coastal areas (Borges et al. 2007). Nonetheless, larval drift via water currents likely represents the predominant mode of dispersal and enables *T. delaisi* to colonize new habitat.

*Tripterygion delaisi* from the eastern Atlantic islands (Azores, Canaries) are genetically divergent from *T. delaisi* in the European Atlantic coast and the Mediterranean, and population differentiation exists between distant locations in the latter areas (Carreras‐Carbonell et al. 2006; Domingues et al. 2007). At a smaller geographic scale, microsatellite markers revealed significant differentiation and a pattern of isolation by distance among populations along the Spanish Mediterranean coast (Carreras‐Carbonell et al. 2006). Except for two individuals from an unspecified location in northern Croatia (Domingues et al. 2007), Adriatic *T. delaisi* have never been examined. Here, we use mitochondrial control region sequences of *T. delaisi* from the north‐eastern Adriatic Sea (Croatia) to analyse the phylogeographic structure within this area and compare them to published sequences to investigate the relationship to *T. delaisi* from other parts of the Mediterranean and from the Atlantic Sea. The sampled area belongs to one of the cyclonic gyres (the middle Adriatic gyre, Fig. 1), within which water currents may provide for substantial dispersal of planktonic larvae. Given the life history of the species and the geomorphological and hydrological features of the Adriatic Sea, we predicted that the sampled populations would show genetic signatures of recent population expansion, only weak phylogeographic structure, but genetic differentiation from their conspecifics in the rest of Mediterranean. Furthermore, we apply an expansion dating approach to provide a first substitution rate estimate of the mitochondrial control region for the suborder Blennioidei.

## Materials and Methods

### Sample collection and DNA sequencing

Eighty‐four individuals of *T. delaisi* were caught with hand nets at 10 localities in Croatia between 2006 and 2012 (Fig. 1, Appendix). Fin clips (small parts of the caudal fin) were taken and preserved in 96% ethanol, and fish were immediately released. Whole genomic DNA was extracted following a rapid Chelex protocol (Richlen and Barber 2005). The most variable part of the mitochondrial control region was amplified and sequenced according to the protocols described in Koblmüller et al. (2011) and Duftner et al. (2005), respectively. The primers used for PCR and chain termination sequencing were L‐Pro‐F_Tropheus (Koblmüller et al. 2011) and TDK‐D (Lee et al. 1995). DNA fragments were purified with Sephadex^™^ G‐50 (GE Healthcare, Vienna, Austria) and visualized on an ABI 3130xl capillary sequencer (Applied Biosystems, Vienna, Austria). Additional sequences from Portugal, Italy, Cyprus, Croatia and the Azores and Canary islands (Domingues et al. 2007) were obtained from GenBank (Fig. 1, Appendix). Sequences were aligned by eye (no indels were present in the alignment) in mega 6.06 (Tamura et al. 2013). The length of the final alignment was 352 bp. Sequences are deposited in GenBank under the accession numbers KT267998–KT268081.

### Phylogeographic analyses

Phylogenetic relationships among haplotypes were inferred by means of a statistical parsimony network (Templeton et al. 1992) and a maximum‐likelihood (ML) tree in tcs 1.2.1 (Clement et al. 2000) and phyml 3.0 (Guindon et al. 2010), respectively. For ML tree search, identical sequences were collapsed into haplotypes using DNACollapser implemented in fabox (Villesen 2007). The HKY+I (Hasegawa et al. 1985) model was employed as best fitting model of sequence evolution selected by the Bayesian information criterion (BIC) in jmodeltest 0.1 (Posada 2008), and statistical support was assessed from 1000 bootstrap replicates.

Haplotype (*H*_d_) and nucleotide diversity (π) were calculated in dnasp 5.10 (Librado and Rozas 2009) for the entire Mediterranean + Atlantic coast samples, as well as for the Croatian samples only. A genetic landscape shape interpolation analysis from the Croatian samples was performed in alleles in space 1.0 (AIS: Miller 2005). The genetic landscape analysis employed a Delaunay triangulation connectivity network and residual genetic distances derived from a linear regression of genetic distances on geographic distances (as recommended for data sets with substantial variation in geographic distances between sampling sites connected in the Delaunay triangulation network; Manni et al. 2004). Grid size was set to 0.05 × 0.05 latitude and longitude degree, respectively, and a distance weighting parameter α = 1 was used (see Miller et al. 2006 for more details on the method). Qualitatively similar results were obtained applying different grid sizes and a range of distance weighting parameters (α = 0.3–3; not shown).

### Substitution rate estimation by expansion dating

To test for signals of past population expansion, we calculated Fu's *F*_S_ (Fu 1997) and Tajima's *D* (Tajima 1989) in dnasp 5.10 and employed a Bayesian coalescent approach [Bayesian skyline plot (BSP)], implemented in beast 1.8.0 (Drummond and Rambaut 2007). For the BSP analysis, we applied the HKY+I model of molecular evolution and a strict molecular clock model. Two independent MCMC runs of ten million generations each were conducted, sampling every 1000th step with a burn‐in of the first 25% of sampled generations. Verification of effective sample sizes (ESS > 200 for all parameters), trace of MCMC runs and visualization of demographic changes were done in tracer 1.5 (available from http://beast.bio.ed.ac.uk/tracer.). The logged parameter values and trees from the two replicate runs were combined using logcombiner 1.8.0 (part of the beast package), and tracer was used to create a BSP.

We are not aware of published substitution rate estimates for the mitochondrial control region in Tripterygiidae, or even the Blennioidei. Rate estimates for closely related perciform families range from 1.8 to >33% per MY (reviewed in Bowen et al. 2006), rendering the selection of a rate for *Tripterygion* into guesswork. Therefore, we used the data produced in this study to derive a substitution rate based on expansion dating. During the LGM (~18 ka BP), the sea level was ~120 m below its present level (Siddall et al. 2003) and large parts of the Adriatic Sea were dry (Trincardi et al. 1994; Lambeck and Purcell 2005). The following deglaciation was associated with a global rise of the sea level, which started gradually and reached a maximum rate at ~14.5 ka BP (Crandall et al. 2012). Shortly thereafter, at around 13 ka BP, surface water temperatures in the Adriatic increased to temperate conditions (Zonneveld 1996). The rising sea levels shifted the Adriatic coastline northwards by ~250 km (Lambeck and Purcell 2005) and greatly expanded the available habitat of *T. delaisi*. Provided that the habitat expansion gave rise to population growth, the palaeohydrologic and palaeoclimatic data suggest that the onset of population growth should have occurred between 18 and 13 ka BP.

The BSP of the Adriatic *T. delaisi* sample indicated a sudden transition from an extended period of constant population size to rapid growth in the more recent past. Therefore, we adopted the expansion dating approach of Crandall et al. (2012) and calibrated the tripterygiid molecular clock based on the assumption that this transition coincided with either the rising sea level or the warming of the surface waters. We applied a two‐epoch coalescent model (Shapiro et al. 2004), as implemented in beast, that simulated a two‐parameter exponential growth (female effective population size scaled by mutation rate, fN_e_μ1; and intrinsic growth rate, *r*) preceded by a one‐parameter constant size model (fN_e_μ0), with a parameter for the transition time between the two epochs (t_transition). We used the same molecular evolution model as for the BSP. 1/x priors were used for all population size parameters, and simple uniform priors were employed for r and t_transition. Upper and lower limits of the prior distribution for each parameter in the model were set using the 95% highest posterior density (HPD) intervals from the BSP as guideline. Run length was 20 million generations, with sampling every 1000th step and a burn‐in of the first 25% of sampled generations (ESS > 200). The mutation rate was calculated as μ = t_transition/*c*, where *c* is the calibration point. Assuming that population growth started either with rising sea levels after the LGM at around 18 ka BP or with surface water warming at 13 ka BP, we estimated μ = t_transition/18 000 to t_transition/13 000.

To explicitly test whether the two‐epoch model indeed fits the data better than a simple constant population size model, we employed a Bayes factors (BFs) approach for model selection (Suchard et al. 2001). Marginal likelihoods for the BF calculation were estimated under both the two‐epoch and the constant population size model by path sampling (PS; Lartillot and Philippe 2006) and stepping stone sampling (SS; Xie et al. 2011), two approaches that were shown to provide accurate and reliable marginal‐likelihood estimates for model comparisons (Baele et al. 2012). PS and SS were conducted in beast (20 million generations, 100 path steps, following a burn‐in of 200 000 generations), adopting the XML codes for PS and SS from Baele et al. (2012, 2013).

## Results

### Phylogeographic pattern

**Figure 2:**
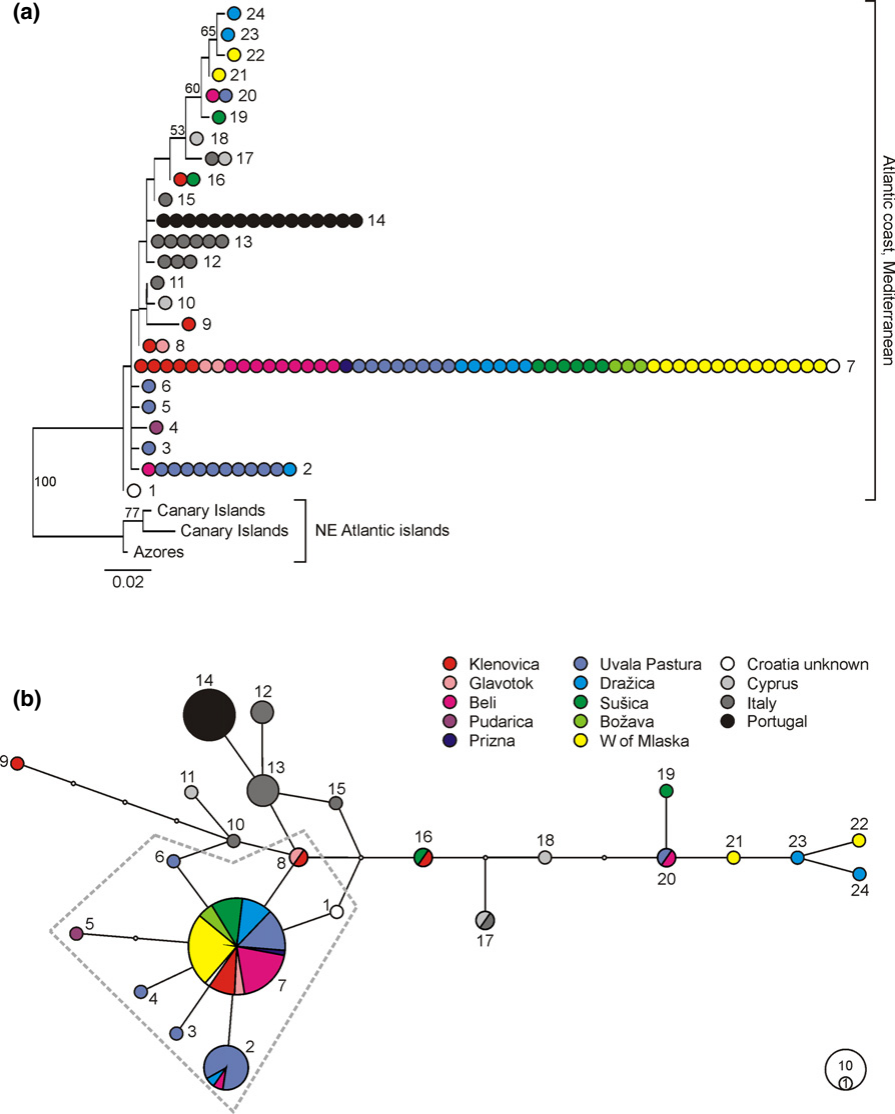
Phylogenetic relationships among mitochondrial control region haplotypes. (a) ML tree based on the HKY‐I model of molecular evolution. Only bootstrap values >50 are shown. (b) Statistical parsimony network. The size of the circles is proportional to the number of samples per haplotype; small empty circles indicate missing haplotypes. The star‐like Adriatic haplogroup is indicated by a hatched line. Numbers next to haplotypes indicate haplotype IDs.

In total, 24 haplotypes were detected in 117 Mediterranean + Atlantic coast samples, which were clearly divergent from the three Atlantic island haplotypes (also see Domingues et al. 2007). The Croatian sample contained 16 different haplotypes, none of which were shared with *T. delaisi* from other parts of the Mediterranean or from the Atlantic (Fig. 2). Genetic landscape shape visualizations revealed no major phylogeographic breaks within Croatia with little genetic distance between sampling localities (Fig. 1c). The darker shading in the area between the islands Cres and Pag suggests that genetic structure is higher among the northern island locations than between these locations and the more southern populations at Ugljan and Hvar.

The majority of the Croatian haplotypes belong to a star‐shaped haplogroup, which is indicative of recent population expansion. Additionally, the Croatian sample contains several more divergent haplotypes at low frequencies. In part owing to these divergent haplotypes, the genetic diversity within the Croatian sample is nearly as high as in the entire Mediterranean + Atlantic coast sample (Croatia, *H*_d_ = 0.544, π = 0.00448; Mediterranean + Atlantic coast, *H*_d_ = 0.734, π = 0.00659). The apparently derived position of the divergent low‐frequency haplotypes in the ML tree (Fig. 2) is most likely an artefact resulting from the low in‐group divergence, the low statistical support for in‐group nodes and from the comparatively large divergence from the out‐group, all of which compromise the accurate positioning of the root (see e.g. Wheeler 1990; Kirchberger et al. 2014).

### Substitution rate estimation by expansion dating

**Figure 3:**
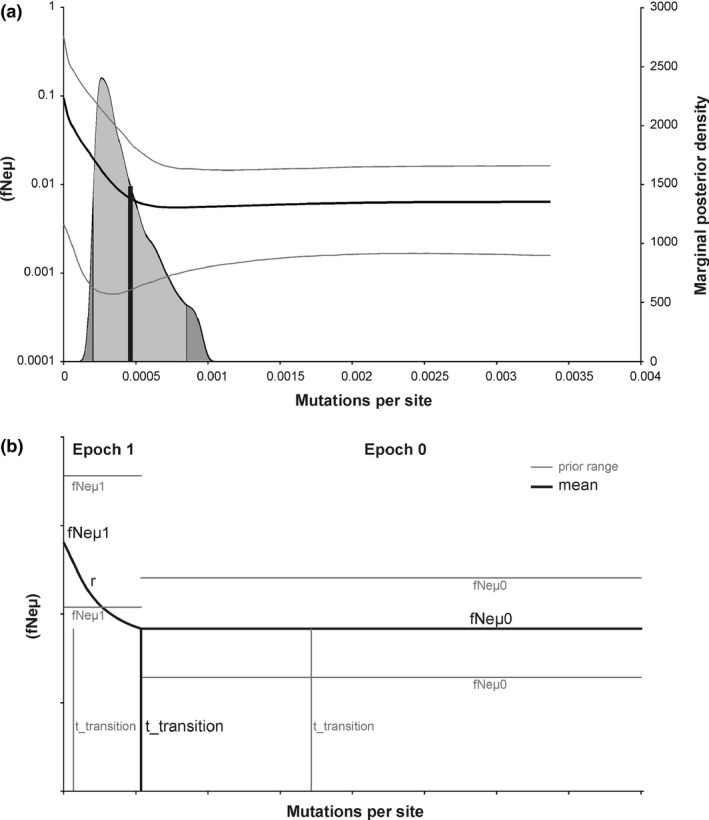
Signatures of population expansion and inference of the substitution rate by expansion dating. (a) Bayesian skyline plot (BSP) for the Adriatic samples. The thick black line depicts the median value for female effective population size (fN_e_μ), and the thin grey lines depict the 95% HPD interval. The marginal posterior distribution for the time of transition (t_transition) from a period of constant population size to recent exponential population growth, inferred from the two‐epoch model and used to calibrate the molecular clock, is overlaid on the BSP with grey shading (posterior density given on the right hand vertical axis). The thick line indicates the mean value of t_transition. Dark shading indicates areas beyond the 95% HPD interval. (b) Schematic illustration of the two‐epoch model employed to estimate t_transition, using the BSP 95% HPDs as priors. r, Exponential growth rate.

Tajima's *D* and Fu's *F*_S_ statistics calculated with the sequences of the full Croatian sample were significantly negative (*D* = −1.6261, p = 0.0257; *F*_S_ = −8.2318, p = 0.0012), which supports the scenario of recent population growth suggested by the star‐shaped part of the haplotype network in Fig. 2. The BSP analysis based on all Croatian haplotypes clearly indicated population growth in the recent past (Fig. 3).

Model selection analysis based on BFs favoured the two‐epoch model with constant population size followed by recent exponential population growth, which simulates the inferred BSP patterns, over a pure constant population size model (Table 1). The 2 ln BF estimates of 6.42 (SS) and 6.6 (PS) indicate strong support for the two‐epoch model (Kass and Raftery 1995). Mean values for the transition time between constant population size and exponential growth were similar to the infliction point depicted in the BSP model (Fig. 3a). Posterior distributions for t_transition, fN_e_μ0, fN_e_μ1 and r were all unimodal. We used the transition time between the constant population size period and the recent phase of exponential growth for calibrating the substitution rate of the analysed control region fragment. The mean parameter estimate for t_transition was 4.6974 × 10^−4^. Assuming that the population expansion in the Adriatic Sea was associated with rising water levels after the LGM about 18 ka BP or with surface water warming at 13 ka BP, this translates into mean estimates of μ of 2.61% (calibrated with water level rise) to 3.61% (calibrated with warming) per site per MY (95% HPD, 1.11–6.77%).

**Table 1:** Demographic model comparison using a model selection approach based on Bayes factors (BFs)

	MLE	
	PS	SS
Two‐epoch model	−759.248*	−759.226*
Constant population size model	−762.457	−762.533
2 ln BF	6.42	6.60

The marginal‐likelihood estimates (MLE) of the two competing models are listed, with the preferred model indicated by an asterisk.

PS, path sampling; SS, stepping stone sampling.

## Discussion

### Phylogeographic pattern

Consistent with expectations based on the life history of *T. delaisi* and the geomorphological and hydrological features of the Adriatic Sea, mitochondrial sequences indicated recent population expansion, extensive haplotype sharing among locations connected by the mid‐Adriatic cyclonic gyre and differentiation from conspecifics in the Mediterranean and the Atlantic. The majority of the Adriatic samples were joined in one single ‘Adriatic’ haplogroup featuring the star‐like shape indicative of population expansion. Additionally, several individuals from various Adriatic locations carried different divergent haplotypes some of which were similar to those sampled in other parts of the Mediterranean (Cyprus, Italy). These divergent haplotypes might either represent polymorphisms dating from the original colonization of the Adriatic, or gene flow from another Adriatic gyre system or other parts of the Mediterranean. While a more thorough sampling from outside the current study area is required to distinguish between these alternatives, gene flow into our study area after its original colonization is certainly a realistic assumption. Moreover, if several divergent haplotypes had been present in the Adriatic prior to the inferred population expansion, it is difficult to explain why only one of them gave rise to a star‐shaped haplogroup, while the others remained at low frequencies.

The phylogeographic pattern in our data relates to the characteristic circulation systems of the Adriatic basin (Astraldi et al. 1999). The Adriatic circulation systems are strongly influenced by wind conditions, Po River discharge and weather conditions in general, which cause seasonal patterns of variation in the flow of water currents (Ursella et al. 2007). Mean currents parallel to the shoreline (eastern Adriatic current, EAC; western Adriatic current, WAC) are weak or almost absent in spring and summer, while cyclonic gyres are present throughout the year in the middle and southern Adriatic, but are particularly well‐developed in spring and summer (Russo and Artegiani 1996; Ursella et al. 2007; see Fig. 1). Reproduction in *T. delaisi* takes place in spring and summer (Wirtz 1978). Consequently, planktonic larvae are present in periods with strong cyclonic circulations in the Adriatic Sea. Along the Portuguese Atlantic coast, larvae of *T. delaisi* appeared to be constrained to inshore regions, and no larvae were found offshore (Borges et al. 2007). Consistently, high levels of self‐recruitment in populations of the Spanish Mediterranean suggested that larvae remain near their natal areas (Carreras‐Carbonell et al. 2007). With their inshore distribution, larvae of *T. delaisi* in the Adriatic may largely escape the offshore water currents, but will still be caught by local, typically variable and reversible currents running near the coast and between the islands (Shanks 2009). The lack of phylogeographic structure in the Adriatic sample certainly indicates that gene flow occurs throughout the investigated area, probably helped by both the middle Adriatic gyre and small local currents. Additionally, the genetic landscape shape analysis identified a slightly increased genetic structure among the northern populations. A possible explanation for this may lie in the sampling of some populations from small and sheltered bays (e.g. Uvala Pastura on Pag), which may experience less gene flow than populations that are more exposed to water currents.

While local currents promote dispersal of *T. delaisi* on smaller geographic scales, any planktonic larvae drifting offshore will probably be trapped in their cyclonic gyre and prevented from dispersal across gyres and beyond the Adriatic basin. Investigations of fine‐scale population structure of organisms within the Adriatic basin are scarce (Maltagliati et al. 2010), but differentiation between populations in the Adriatic Sea and the rest of the Mediterranean, as observed in *T. delaisi*, has also been reported for several other organisms (e.g. planktonic chaetognaths, *Sagitta setosa*: Peijnenburg et al. 2004; common shrimp, *Crangon crangon*: Luttikhuizen et al. 2008; edible sea urchin, *Paracentrotus lividus*: Maltagliati et al. 2010; red mullet, *Mullus barbatus*: Maggio et al. 2009; Mediterranean rainbow wrasse, *Coris julis*: Fruciano et al. 2011; marbled goby, *Pomatoschistus minutus*: Mejri et al. 2011; small‐spotted catshark, *Scyliorhinus canicula*: Gubili et al. 2014). Notably, all these taxa are poor active dispersers and/or rely on passive dispersal via water currents in planktonic life stages (or are planktonic throughout their live). In contrast, highly mobile, actively dispersing taxa display no or only very little phylogeographic structure across the entire Mediterranean (e.g. bonito, *Sarda sarda*: Viñas et al. 2004; mackerel, *Scomber scombrus*: Zardoya et al. 2004; anchovy, *Engraulis encrasicolus*: Zarraonaindia et al. 2012).

### Expansion dating

Population expansion in the wake of rising sea levels following the lowstand during the LGM has been reported for numerous marine species (e.g. Marko et al. 2010; Naro‐Maciel et al. 2011; Crandall et al. 2012; Ho et al. 2014), including some species from the Adriatic Sea (e.g. Debes et al. 2008; Luttikhuizen et al. 2008). Given that Adriatic *T. delaisi* also display genetic signatures of population expansion, we took advantage of the likely concomitance of population growth with the postglacial habitat expansion and applied an expansion dating approach to estimate the substitution rate of the mitochondrial control region in Trypterigiidae. Using the period between the LGM and the warming of surface waters as probable onsets of population expansion (18–13 ka BP), we estimated the substitution rate based on a two‐epoch coalescent model in beast as 2.61–3.61% per site per MY (95% HPD, 1.11–6.77%). Our rate estimates fall within the range of control region substitution rates estimated from and applied to other perciform fishes, for example Pseudochromidae (3.2% per site per MY; Messmer et al. 2005), Pomacentridae (3.47–3.92% per site per MY; Domingues et al. 2005) and Cichlidae (3.24–5.7% per site per MY; e.g. Koblmüller et al. 2009; Genner et al. 2010). In contrast, the range of substitution rate estimates previously used in studies of blennioids, including Tripterygiidae, is comparatively wide and ranges from 1.8 to 10% per site per MY (e.g. Domingues et al. 2007; Hickey et al. 2009; Francisco et al. 2011). These estimates were derived from the trans‐Isthmian divergence of two closely related pomacentrid fishes (Domingues et al. 2005) in one case, and adopted from data of galaxiid fish in New Zealand (Waters and Burridge 1999; Burridge et al. 2008) in another. The internally calibrated estimates obtained in the present study are likely an improvement over values borrowed from distant species. Due to the time dependency of molecular rates (Burridge et al. 2008; Peterson and Masel 2009; Ho et al. 2011; but see Emerson and Hickerson 2015), the very recent calibration points used in our dating restrict the applicability of our substitution rate estimates to analyses of recent evolution, in the scales of tens and perhaps hundreds of thousands of years.

## Concluding remarks

The present study is one of only a few to address the genetic structure of animals within the Adriatic basin. High rates of gene flow, likely mediated by planktonic dispersal, were inferred among locations within a cyclonic gyre. Further sampling across gyres will be required to test whether for passive dispersers, the cyclonic gyres partition the Adriatic basin into discrete phylogeographic regions, or whether boundaries between gyres can be overcome with the help of local inshore currents. The phylogeographic separation of the Adriatic Sea from the rest of the Mediterranean, which has been observed in a range of taxa with limited active dispersal abilities, is also evident in the data produced and used in the current study. Finally, we report a novel substitution rate for our study species, which will facilitate future phylogenetic and population genetic work within and beyond Tripterygiidae.

## Appendix 1 Sequences used in the present study, with sample IDs, information on sampling sites (with coordinates, if available), haplotype ID, reference to the original study that generated the DNA sequences, and GenBank accession numbers

**Table:**

Sample ID^a^	Sampling site	Coordinates	Haplotype^b^	GenBank Acc. Nr.	Reference
BeliCres2F10	Beli, Cres, Croatia	45.11°N, 14.36°E	7	KT267998	This study
BeliCres2F6	Beli, Cres, Croatia	45.11°N, 14.36°E	7	KT267999	This study
BeliCres2G1	Beli, Cres, Croatia	45.11°N, 14.36°E	7	KT268000	This study
BeliCres2G10	Beli, Cres, Croatia	45.11°N, 14.36°E	20	KT268001	This study
BeliCres2G5	Beli, Cres, Croatia	45.11°N, 14.36°E	2	KT268002	This study
BeliCres2G7	Beli, Cres, Croatia	45.11°N, 14.36°E	7	KT268003	This study
BeliCres2G8	Beli, Cres, Croatia	45.11°N, 14.36°E	7	KT268004	This study
BeliCres2G9	Beli, Cres, Croatia	45.11°N, 14.36°E	7	KT268005	This study
BeliCres2H6	Beli, Cres, Croatia	45.11°N, 14.36°E	7	KT268006	This study
BeliCres2I2	Beli, Cres, Croatia	45.11°N, 14.36°E	7	KT268007	This study
BeliCres2I3	Beli, Cres, Croatia	45.11°N, 14.36°E	7	KT268008	This study
BeliCresK061E10	Beli, Cres, Croatia	45.11°N, 14.36°E	7	KT268009	This study
BeliCresK061E6	Beli, Cres, Croatia	45.11°N, 14.36°E	7	KT268010	This study
BozavaDugiOtok2B9	Božava, Dugi Otok, Croatia	44.14°N, 14.91°E	7	KT268011	This study
BozavaDugiOtok2D9	Božava, Dugi Otok, Croatia	44.14°N, 14.91°E	7	KT268012	This study
BozavaDugiOtokK061C1	Božava, Dugi Otok, Croatia	44.14°N, 14.91°E	7	KT268013	This study
UvalaPastorePag1	Uvala Pastore, Pag, Croatia	44.60°N, 14.84°E	3	KT268014	This study
UvalaPastorePag10	Uvala Pastore, Pag, Croatia	44.60°N, 14.84°E	2	KT268015	This study
UvalaPastorePag11	Uvala Pastore, Pag, Croatia	44.60°N, 14.84°E	2	KT268016	This study
UvalaPastorePag12	Uvala Pastore, Pag, Croatia	44.60°N, 14.84°E	6	KT268017	This study
UvalaPastorePag13	Uvala Pastore, Pag, Croatia	44.60°N, 14.84°E	7	KT268018	This study
UvalaPastorePag14	Uvala Pastore, Pag, Croatia	44.60°N, 14.84°E	2	KT268019	This study
UvalaPastorePag15	Uvala Pastore, Pag, Croatia	44.60°N, 14.84°E	7	KT268020	This study
UvalaPastorePag16	Uvala Pastore, Pag, Croatia	44.60°N, 14.84°E	2	KT268021	This study
UvalaPastorePag17	Uvala Pastore, Pag, Croatia	44.60°N, 14.84°E	2	KT268022	This study
UvalaPastorePag18	Uvala Pastore, Pag, Croatia	44.60°N, 14.84°E	7	KT268023	This study
UvalaPastorePag19	Uvala Pastore, Pag, Croatia	44.60°N, 14.84°E	2	KT268024	This studyv
UvalaPastorePag20	Uvala Pastore, Pag, Croatia	44.60°N, 14.84°E	2	KT268025	This study
UvalaPastorePag22	Uvala Pastore, Pag, Croatia	44.60°N, 14.84°E	2	KT268026	This study
UvalaPastorePag23	Uvala Pastore, Pag, Croatia	44.60°N, 14.84°E	2	KT268027	This study
UvalaPastorePag24	Uvala Pastore, Pag, Croatia	44.60°N, 14.84°E	7	KT268028	This study
UvalaPastorePag25	Uvala Pastore, Pag, Croatia	44.60°N, 14.84°E	7	KT268029	This study
UvalaPastorePag27	Uvala Pastore, Pag, Croatia	44.60°N, 14.84°E	20	KT268030	This study
UvalaPastorePag4	Uvala Pastore, Pag, Croatia	44.60°N, 14.84°E	4	KT268031	This study
UvalaPastorePag6	Uvala Pastore, Pag, Croatia	44.60°N, 14.84°E	2	KT268032	This study
UvalaPastorePag7	Uvala Pastore, Pag, Croatia	44.60°N, 14.84°E	7	KT268033	This study
UvalaPastorePag8	Uvala Pastore, Pag, Croatia	44.60°N, 14.84°E	7	KT268034	This study
UvalaPastorePag9	Uvala Pastore, Pag, Croatia	44.60°N, 14.84°E	7	KT268035	This study
Prizna6	Prizna, Croatia	44.62°N, 14.96°E	7	KT268036	This study
GlavotokKrk18	Glavotok, Krk, Croatia	45.13°N, 14.53°E	8	KT268037	This study
GlavotokKrk2	Glavotok, Krk, Croatia	45.13°N, 14.53°E	7	KT268038	This study
GlavotokKrk28	Glavotok, Krk, Croatia	45.13°N, 14.53°E	7	KT268039	This study
Klenovica1A8	Klenovica, Croatia	45.10°N, 14.84°E	7	KT268040	This study
Klenovica1B10	Klenovica, Croatia	45.10°N, 14.84°E	7	KT268041	This study
Klenovica1B9	Klenovica, Croatia	45.10°N, 14.84°E	7	KT268042	This study
Klenovica1C1	Klenovica, Croatia	45.10°N, 14.84°E	8	KT268043	This study
Klenovica1C2	Klenovica, Croatia	45.10°N, 14.84°E	9	KT268044	This study
Klenovica1C4	Klenovica, Croatia	45.10°N, 14.84°E	7	KT268045	This study
KlenovicaK061A2	Klenovica, Croatia	45.10°N, 14.84°E	7	KT268046	This study
KlenovicaK061A5	Klenovica, Croatia	45.10°N, 14.84°E	16	KT268047	This study
Mlaska1_1	W of Mlaska, Croatia	43.13°N, 17.11°E	7	KT268048	This study
Mlaska1_2	W of Mlaska, Croatia	43.13°N, 17.11°E	7	KT268049	This study
Mlaska1_3	W of Mlaska, Croatia	43.13°N, 17.11°E	7	KT268050	This study
Mlaska1_4	W of Mlaska, Croatia	43.13°N, 17.11°E	7	KT268051	This study
Mlaska1_5	W of Mlaska, Croatia	43.13°N, 17.11°E	7	KT268052	This study
Mlaska2_1	W of Mlaska, Croatia	43.13°N, 17.11°E	7	KT268053	This study
Mlaska2_2	W of Mlaska, Croatia	43.13°N, 17.11°E	7	KT268054	This study
Mlaska2_10	W of Mlaska, Croatia	43.13°N, 17.11°E	7	KT268055	This study
Mlaska2_3	W of Mlaska, Croatia	43.13°N, 17.11°E	21	KT268056	This study
Mlaska2_4	W of Mlaska, Croatia	43.13°N, 17.11°E	7	KT268057	This study
Mlaska2_5	W of Mlaska, Croatia	43.13°N, 17.11°E	7	KT268058	This study
Mlaska2_6	W of Mlaska, Croatia	43.13°N, 17.11°E	7	KT268059	This study
Mlaska2_7	W of Mlaska, Croatia	43.13°N, 17.11°E	7	KT268060	This study
Mlaska2_8	W of Mlaska, Croatia	43.13°N, 17.11°E	7	KT268061	This study
Mlaska2_9	W of Mlaska, Croatia	43.13°N, 17.11°E	7	KT268062	This study
Mlaska3_1	W of Mlaska, Croatia	43.13°N, 17.11°E	22	KT268063	This study
PagW1C5	Dražica, Pag, Croatia	44.63°N, 14.80°E	2	KT268064	This study
PagW1D3	Dražica, Pag, Croatia	44.63°N, 14.80°E	23	KT268065	This study
PagW1D9	Dražica, Pag, Croatia	44.63°N, 14.80°E	7	KT268066	This study
PagW1E2	Dražica, Pag, Croatia	44.63°N, 14.80°E	7	KT268067	This study
PagW1E7	Dražica, Pag, Croatia	44.63°N, 14.80°E	7	KT268068	This study
PagW1E8	Dražica, Pag, Croatia	44.63°N, 14.80°E	24	KT268069	This study
PagW1E9	Dražica, Pag, Croatia	44.63°N, 14.80°E	7	KT268070	This study
PagWK061A6	Dražica, Pag, Croatia	44.63°N, 14.80°E	7	KT268071	This study
PagWK061A8	Dražica, Pag, Croatia	44.63°N, 14.80°E	7	KT268072	This study
PudaricaRab10	Pudarica, Rab, Croatia	44.72°N, 14.82°E	5	KT268073	This study
UgljanEK06A9	Sušica, Ugljan, Croatia	44.14°N, 15.09°E	7	KT268074	This study
UgljanEK061B3	Sušica, Ugljan, Croatia	44.14°N, 15.09°E	7	KT268075	This study
UgljanO1F10	Sušica, Ugljan, Croatia	44.14°N, 15.09°E	7	KT268076	This study
UgljanO1F5	Sušica, Ugljan, Croatia	44.14°N, 15.09°E	16	KT268077	This study
UgljanO1G9	Sušica, Ugljan, Croatia	44.14°N, 15.09°E	7	KT268078	This study
UgljanO2A2	Sušica, Ugljan, Croatia	44.14°N, 15.09°E	7	KT268079	This study
UgljanOK061A10	Sušica, Ugljan, Croatia	44.14°N, 15.09°E	19	KT268080	This study
UgljanOK061B2	Sušica, Ugljan, Croatia	44.14°N, 15.09°E	7	KT268081	This study
Arr1	Arrábida, Portugal	–	14	EF484571	Domingues et al. (2007)
Arr2	Arrábida, Portugal	–	14	EF484572	Domingues et al. (2007)
Arr3	Arrábida, Portugal	–	14	EF484573	Domingues et al. (2007)
Arr4	Arrábida, Portugal	–	14	EF484574	Domingues et al. (2007)
Arr5	Arrábida, Portugal	–	14	EF484575	Domingues et al. (2007)
Arr6	Arrábida, Portugal	–	14	EF484576	Domingues et al. (2007)
Arr7	Arrábida, Portugal	–	14	EF484577	Domingues et al. (2007)
Arr8	Arrábida, Portugal	–	14	EF484578	Domingues et al. (2007)
Arr9	Arrábida, Portugal	–	14	EF484579	Domingues et al. (2007)
Arr10	Arrábida, Portugal	–	14	EF484580	Domingues et al. (2007)
Arr11	Arrábida, Portugal	–	14	EF484581	Domingues et al. (2007)
Arr12	Arrábida, Portugal	–	14	EF484582	Domingues et al. (2007)
Arr13	Arrábida, Portugal	–	14	EF484583	Domingues et al. (2007)
Arr14	Arrábida, Portugal	–	14	EF484584	Domingues et al. (2007)
Arr19	Arrábida, Portugal	–	14	EF484585	Domingues et al. (2007)
Arr20	Arrábida, Portugal	–	14	EF484586	Domingues et al. (2007)
Ita1	Capri, Italy	–	13	EF484587	Domingues et al. (2007)
Ita2	Capri, Italy	–	10	EF484588	Domingues et al. (2007)
Ita3	Capri, Italy	–	13	EF484589	Domingues et al. (2007)
Ita4	Vivara, Italy	–	17	EF484590	Domingues et al. (2007)
Ita5	Vivara, Italy	–	12	EF484591	Domingues et al. (2007)
Ita6	Vivara, Italy	–	12	EF484592	Domingues et al. (2007)
Ita7	Vivara, Italy	–	12	EF484593	Domingues et al. (2007)
Ita8	Vivara, Italy	–	13	EF484594	Domingues et al. (2007)
Ita9	Vivara, Italy	–	13	EF484595	Domingues et al. (2007)
Ita10	Vivara, Italy	–	13	EF484596	Domingues et al. (2007)
Ita11	Vivara, Italy	–	13	EF484597	Domingues et al. (2007)
Ita12	Vivara, Italy	–	15	EF484598	Domingues et al. (2007)
Cro1	Croatia	–	7	EF484599	Domingues et al. (2007)
Cro2	Croatia	–	1	EF484600	Domingues et al. (2007)
Cyp1	Cyprus	–	11	EF484601	Domingues et al. (2007)
Cyp3	Cyprus	–	18	EF484602	Domingues et al. (2007)
Cyp4	Cyprus	–	17	EF484603	Domingues et al. (2007)
Azo103	Azores	–	–	EF484541	Domingues et al. (2007)
Can10	Canary Islands	–	–	EF484563	Domingues et al. (2007)
Can12	Canary Islands	–	–	EF484565	Domingues et al. (2007)

a Sample IDs refer to IDs of fin clips and DNA extracts, which are stored at the Institute of Zoology, University of Graz.

b Haplotype numbers have been assigned to the Mediterranean (including Atlantic coast) samples only.
